# Influence of Personal Protective Equipment on Wildland Firefighters’ Physiological Response and Performance during the Pack Test

**DOI:** 10.3390/ijerph18105050

**Published:** 2021-05-11

**Authors:** Belén Carballo-Leyenda, Jorge Gutiérrez-Arroyo, Fabio García-Heras, Pilar Sánchez-Collado, José G. Villa-Vicente, Jose A. Rodríguez-Marroyo

**Affiliations:** VALFIS Research Group, Institute of Biomedicine, University of León, 24071 León, Spain; jgutia@unileon.es (J.G.-A.); fgarh@unileon.es (F.G.-H.); mpsanc@unileon.es (P.S.-C.); jg.villa@unileon.es (J.G.V.-V.)

**Keywords:** wildland firefighting, personal protective equipment, readiness for work, physical employment standards, work capacity

## Abstract

The Pack Test (PT) is a widely used test to establish readiness for work in wildland firefighting. It is common to perform this test dressed in regular exercise clothing. However, wildland firefighters (WFF) have to wear personal protective equipment (PPE) during their deployments, which increases the physiological strain and reduces their work capacity. This study aimed to analyse the impact of full PPE on PT performance. Nine male professional WFF performed in random order a PT walking at the fastest possible self-pace wearing two different clothing configurations: (i) traditional short sports gear (SG) and (ii) the PPE currently used by Spanish WFF. Heart rate (HR), rating of perceived exertion and lap time were recorded during the PT. In addition, oxygen uptake (VO_2_) was estimated through the individual VO_2_–HR relationship previously obtained during a graded exercise test. There was a significant decrease in the PT performance (i.e., completion time) (~12%, *p* < 0.05) in PPE. The physiological demands with this configuration were significantly higher (~10%, *p* < 0.05). WFF spent ~13 min above the anaerobic threshold in PPE vs. ~4 min in SG. A multiple stepwise regression analysis revealed that PT performance variation might be explained by the maximal aerobic velocity (84.5%) in PPE and the VO_2max_ (75.9%) in SG. In conclusion, wearing complete PPE increases WFF’s physiological strain, which translates into a significant PT performance reduction. Performing the test walking at the fastest possible self-pace wearing the PPE would better reflect the high-intensity effort periods reported in real scenarios.

## 1. Introduction

Fighting wildfire is a physically demanding occupation [1,2] requiring firefighters to be physically fit to minimise fatigue and work safely and competently [3]. To determine whether personnel are fit for duty, an increasing number of firefighting agencies (e.g., the USDA Forest Service; Australian Fire Agencies; British Columbia Forest Service in Canada or Ministry of Agriculture, Fisheries and Food in Spain) employ physical competency tests, such as the Pack Test (PT) [4,5]. This test involves a 4.83 km hike over level terrain while carrying a 20.4 kg pack within 45 min [6]. The PT was designed to challenge an individual’s muscular strength and cardiorespiratory fitness, mimicking the physiological strain encountered during wildland fire suppression using hand tools. While the PT is widely used to assess the subjects’ ability to perform sustained and arduous work [7], some aspects of its protocol are not standardised, which might affect its validity and reliability [8]. Specifically, it is common to perform the PT dressed in sports clothing (e.g., shorts, T-shirt, running shoes). However, wildland firefighters (WFF) have to wear their personal protective equipment (PPE) (i.e., coveralls, gloves, helmet and boots) during their deployments [9,10]. This fact might affect PT performance since wearing a protective ensemble is heavier (~6 kg) and more restrictive to heat dissipation than exercise clothing [11,12].

In this context, the effect of the PPE on physiological strain and work capacity has been well documented [10,13,14,15,16]. It has been recently reported that wearing WFF’s personal protective clothing leads to subjects’ decreased performance (i.e., time to exhaustion) of 17% [10]. Moreover, when other PPE elements such as the helmet, gloves and boots were added, a reduction of 50% in performance was found [10]. In this regard, the important role of the firefighters’ boots has also been previously reported [16]. An increase in exercise demands of approximately 12% for every 1 kg of added mass has been shown. To our knowledge, only Phillips et al. [8] have tried to determine the effect of protective equipment on PT performance in healthy subjects. These authors noted that lightweight boots and a fire-resistant cotton coverall might reduce PT performance by ~10%. Researchers pointed out that these elements should be included when assessing WFF’s fitness, as they help to replicate the workplace-specific demands. However, the findings of this study might have been conditioned by the subjects’ characteristics. A better aerobic capacity (56.2 ± 0.9 mL·kg^−1^·min^−1^) has been reported in professional WFF [9]. Additionally, it might be expected that professional WFF would have a greater adaptation to PPE use. On the other hand, in this study, PPE elements such as the helmets, gloves or neck shield were not used, which might have reduced the subjects’ physiological strain [10]. Therefore, the present study aimed to analyse the impact of complete personal protective equipment (i.e., coverall, gloves, helmet and boots) on Pack Test performance in professional wildland firefighters.

## 2. Material and Methods

### 2.1. Participants

Nine male professional wildland firefighters participated in this study (Table 1). All of them had more than 7 years of experience as elite wildland firefighters and performed endurance exercise (45–60 min per training session) 3 times per week as part of their scheduled training. Written informed consent was obtained from the participants before starting the study. The test protocol was developed according to the Declaration of Helsinki guidelines for research on human subjects, and it was approved by the Ethics Committee of the University of León, Spain.

### 2.2. Experimental Design

For 2 weeks, subjects performed 3 testing sessions on separate days, with at least 72 h in between. Participants were asked to refrain from strenuous exercise, excessive sun exposure and alcohol consumption for 48 h before each experimental session. Volunteers were asked to consume ~250 mL of water 1 h before beginning each experimental session [17]. A graded exercise test was performed during the first testing session to determine the subjects’ maximal aerobic capacity (VO_2max_). During the second and third training sessions, participants performed the PT in random order wearing 2 different clothing configurations: (i) traditional short sports gear (SG) (i.e., shorts, cotton t-shirt, underwear, cotton socks and running shoes) and (ii) the personal protective equipment (PPE) currently used by Spanish WFF (Madrid, Spain), which includes thermal-resistant clothing (65% fire retardant viscose, 30% Nomex and 5% Kevlar, 1.5 kg, surface mass 270 g ± m^−2^, thermal resistance 0.019 m^2^ K·W^−1^ and evaporative resistance 3.79 m^2^·Pa·W^−1^) and other protective elements such as a helmet, neck shroud, gloves, goggles and midcalf leather boots. In this configuration, SG was worn underneath the PPE.

#### 2.2.1. Graded Exercise Test

WFF underwent a graded exercise test on a treadmill (h/p/cosmos pulsar, Cosmos Sports & Medical GMBH, Nussdorf-Traunstein, Germany). The test started at 6 km·h^−1^, and velocity was increased 1 km·h^−1^ every 1 min until volitional exhaustion. The treadmill grade was kept at 1% throughout the test. Respiratory gas exchange was continuously recorded via a breath-by-breath system (Medisoft Ergocard, Medisoft Group, Sorinnes, Belgium) calibrated following the manufacturer’s guidelines. Heart rate (HR) response was also measured with a 12-lead electrocardiogram (Medisoft Medcard, Medi-soft Group, Sorinnes, Belgium). VO_2max_ and maximal HR were recorded as the highest values obtained for the last 30 s period before exhaustion. VO_2max_ was considered as valid when at least 2 of the following criteria were reached [18]: VO_2_ plateau (≤150 mL·min^−1^), RER ≥ 1.15, maximal HR was ±10 beats of the age-predicted maximal HR (220-age) and rating of perceived exertion (CR 0–10) ≥ 8. The maximal velocity was determined as the highest velocity subjects might maintain for a complete stage, plus the interpolated velocity from incomplete stages [19]. The ventilatory (VT) and respiratory compensation threshold (RCT) were identified according to the following criteria [20]: increase in both ventilation equivalent for oxygen (VE·VO_2_^−1^) and end-tidal partial pressure of oxygen with no concomitant increase in ventilation equivalent for carbon dioxide (VE·VCO_2_^−1^) for VT, an increase in both VE·VO_2_^−1^ and VE·VCO_2_^−1^ and a decrease in end-tidal partial pressure of carbon dioxide for RCT.

#### 2.2.2. Pack Test

The PTs were performed on an outdoor 400 m running track at the same time of day under similar environmental conditions (14.6 ± 1.9 °C and 75.0 ± 14.0% relative humidity). After a standardized warm-up (15 min of submaximal running and free stretching for 5 min), WFF had to complete 12 full laps for a total distance of 4.8 km in less than 45 min, while carrying a correctly fitted 75 L backpack weighted to 20.4 kg [5,6,7,21]. All subjects were instructed to complete the test as fast as possible without running and to stop after completing the required distance. Verbal encouragement was provided throughout the test, but no pacing feedback was administered at any point. Subjects were only informed when half of the test was completed. Throughout the trial, the HR response was measured every 5 s (RS800, Polar Electro Oy, Kempele, Finland) and was averaged every 30 s for their analysis. In addition, oxygen uptake (VO_2_) during the PT was estimated through the individual linear relationship between VO_2_ and HR obtained during the graded exercise test [22]. Relative VO_2_ was normalized to total mass, taking into account body mass and load carriage (i.e., backpack and PPE mass) (Taylor et al., 2012). Rating of perceived exertion (RPE) was obtained during the last 10 m of every lap using the Borg scale (1–10) [23]. A photocell timing system (DSD Laser System, DSD Inc., León, Spain) was used to measure the total time and lap time. Finally, walking cadence was calculated by visually counting the number of steps in 1 min in the middle course of laps 1, 6 and 12 [24]. All subjects were previously familiar with the use of the PT.

### 2.3. Data Analysis

The results are expressed as mean ± standard deviation (SD). The assumption of normality was verified using the Shapiro–Wilk’s test. A paired student’s *t*-test was applied to establish differences in PT completion time and walking cadence. The variables analysed throughout the PT (i.e., lap time, HR, %HR_max_, VO_2_ and RPE) were compared using a 2-way ANOVA with repeated measures (clothing configuration × time). When a significant *F*-value was found, Bonferroni’s post hoc comparison was used to establish significant differences between means. The assumption of sphericity was checked using Mauchly’s test, and if this assumption was violated, the Greenhouse–Geisser adjustment was performed. Cohen’s *d* and partial eta-squared (η_p_^2^) were calculated as a measure of effect size. Cohen’s *d* values of <0.20, 0.20–0.50, 0.51–0.80 and >0.80 were rated as trivial, small, moderate and large effects, respectively [25]. When (η_p_^2^) was reported, values of 0.01, 0.06 and 0.14 were considered small, moderate and large, respectively [26]. The relationship between variables was determined using the Pearson correlation coefficient (*r*). Magnitudes of <0.1, <0.4, <0.6, <0.9 and >0.9 were classified as trivial, small, moderate, strong, very strong and almost perfect, respectively [27]. Finally, a multiple stepwise regression analysis was used to establish a predictive equation to estimate subjects’ PT performance from the submaximal and maximal variables assessed in the graded exercise test. Collinearity tolerance statistics were calculated to determine the correlation between predictor variables. Any variable that had a tolerance level of less than 0.10 was not included in the model. Values of *p* < 0.05 were considered statistically significant. Analyses were performed using SPSS+ V.25.0 statistical software (SPSS, Inc., Chicago, IL, USA).

## 3. Results

**Figure 1:**
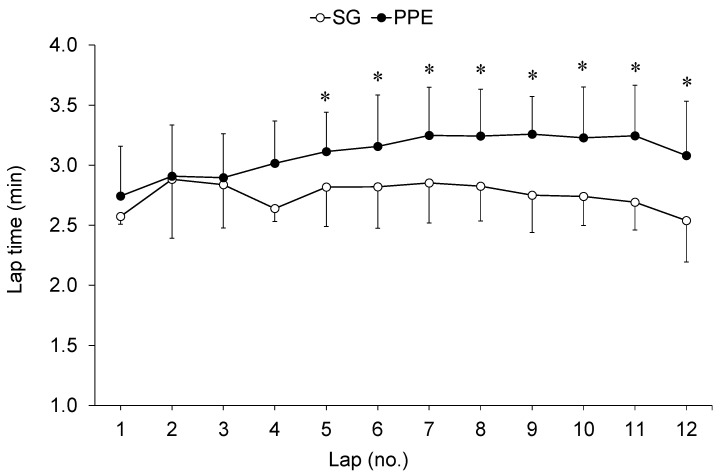
Lap time during the Pack Test performed wearing traditional short sports gear (SG) and wildland firefighters’ personal protective equipment (PPE). Values are mean ± SD. * indicates significant difference (*p* < 0.05).

**Figure 2:**
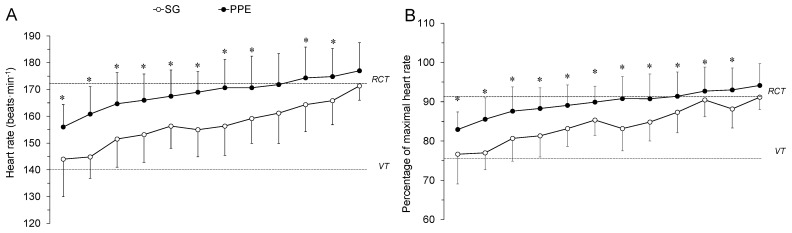

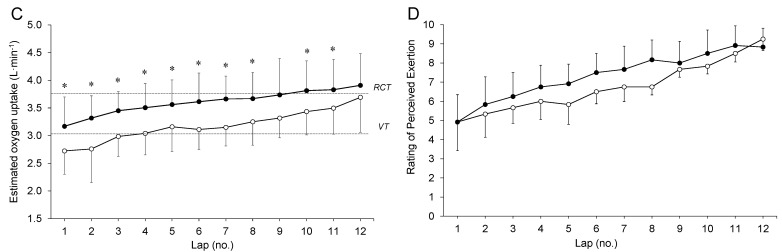
Heart rate (**A**,**B**), estimated VO_2_ (**C**) and rating of perceived exertion (**D**) throughout the Pack Test wearing traditional short sports gear (SG) and a wildland firefighters’ personal protective equipment (PPE). RCT, respiratory compensation threshold; VT, ventilatory threshold. Values are mean ± SD. * indicates significant difference (*p* < 0.05).

The results of the anthropometric and graded exercise tests performed in the laboratory that define the WFF’s morphological and cardiopulmonary characteristics are shown in Table 1. There was a significant decrease in PT performance (*p* < 0.05, *d* = 0.94) when WFF wore PPE (Table 2). A significant (*p* < 0.05) interaction effect between clothing configuration and time was analysed for lap time. From the fifth time lap on, significant differences (*p* < 0.05, η_p_^2^ = 0.45) between PPE and SG were obtained (Figure 1). Physiological demands were significantly higher (*p* < 0.05) in PPE configuration (Table 2). In addition, an interaction effect (*p* < 0.05) between clothing configuration and time on HR and VO_2_ was obtained. Both the HR and estimated VO_2_ were higher with PPE than SG (*p* < 0.05, η_p_^2^ = 0.73, 0.71) throughout the PT (Figure 2). However, no significant differences between conditions in RPE or walking cadence were found (Table 2). Despite this, the magnitude of the differences in the RPE was moderate. Finally, all variables showed a significantly increase over the PT course (*p* < 0.05, η_p_^2^ = 0.54–0.99) (Figure 1 and Figure 2).

There was a strong relationship between PT performance in SG and PPE (*r* = 0.90, *p* < 0.05). Furthermore, the PT performance was correlated with VO_2max_, maximal velocity and velocity at RCT in SG (*r* = −0.90, −0.89 and −0.82, respectively) and PPE (*r* = −0.91, −0.94 and −0.86, respectively). The multiple regression analysis revealed that 84.5% of the PT performance variation might be explained by the maximal aerobic velocity reached in the graded exercise test in PPE (y = 60.89–1.396 maximal velocity, *p* < 0.01, SEE = 1.54 min). Conversely, 75.9% of the PT performance variation might be explained by the VO_2max_ in SG (y = 53.623–0.397 VO_2max_, *p* < 0.05, SEE = 1.73 min).

## 4. Discussion

The main finding of this study was that the use of PPE increased the WFF’s physiological strain (~10%), leading to a significant reduction in PT performance (~12%) versus SG. These results highlight the influence that PPE has on the exercise demands during the PT, which should be taken into account when administering this test to specifically assess the WFF‘s physical competency. Overall, these results are consistent with those previously reported [10,16,28,29], which demonstrate the negative impact on performance when exercising in occupational protective ensembles. The higher physiological demand for PPE (Figure 2) might have been a consequence of the mass difference (6.8 ± 1.4 kg) between PPE and SG [16,28,29]. In this regard, increases in VO_2_ of 2.7% per kg of clothing mass has been previously reported [28]. In addition, mass distribution might have played an important role. In this sense, Taylor et al. [16] showed the importance of the firefighting boots’ mass (~2.5 kg) in increasing metabolic cost (~11%). Therefore, it seems plausible to think that the WFF’s boots in this study (~2.0 kg) might have contributed to the increased HR and estimated VO_2_ (Figure 2).

Although the increase of the mass in PPE might mainly explain the increase in the metabolic rate analysed, the use of the protective suit involves wearing an additional layer, which might suppose a worsened economy of movement [8]. When the estimated VO_2_ was normalised to total mass, a significantly higher value in PPE was found (Table 2). Earlier studies have reported a 3% increase in VO_2_ for each additional layer worn during whole-body exercise [30,31]. Even a reduction in the subjects’ mobility caused by the PPE elements might contribute to this fact by causing a negative effect on PT walking velocity [32].

On the other hand, performing the PT with PPE might lead to greater thermal stress [33,34]. Wearing PPE has been associated with a higher cardiovascular strain due to the body heat dissipation restriction [10,13,14]. This fact forces the thermoregulatory system to increase the cutaneous blood flow in an attempt to enhance body heat release [10,13,14]. In this study, the thermal state between conditions could not be discussed since the core temperature was not measured. However, it may be speculated that the higher HR in PPE (~12 bpm) might, in part, be an added effect of a higher body heat storage as a result of the decrease in the exposed skin surface, reducing heat exchange with the ambient [12,35]. Recently, the role of wearing complete PPE vs. sports clothing on WFF’s thermophysiological response has been reported [10]. Results of Carballo-Leyenda et al. [10] showed that PPE might significantly worsen WFF’s performance and substantially increase physiological demands (~22%) compared to a sports gear ensemble due to both the mass increase and body heat storage.

The findings of the present study substantiate those reported by Phillips et al. [8] These authors informed of a decrease in PT performance of ~3% when healthy subjects wore protective clothing and boots. However, the negative impact of PPE on PT performance was four times higher in our study (~12%). Similarly, the effect that PPE had on HR was greater in our WFF sample (8% vs. 4%). These differences might be related to the fact that our study subjects performed the test completely encapsulated, while subjects in Phillips et al. [8] only wore protective clothing and work boots. Several studies have reported an increase in thermal and cardiovascular strain when covering the head with a helmet, mask or hood [10,16,35,36]. Specifically, the relevance of the PPE elements has been examined. Carballo-Leyenda et al. [10] have shown that adding the helmet, boots and gloves to the fire-resistant coverall may reduce WFF’s performance by 50% and increase the metabolic demand by 20% vs. wearing only the protective suit. Despite this, PT performance was better (~12%) in our study subjects, possibly because of their better aerobic capacity (~56 vs. ~47 mL·kg^−1^·min^−1^). Indeed, the PT performance with a similar configuration (SG) was ~19% better in our subjects. We found a strong relationship (*r* < −0.90) between aerobic capacity parameters (e.g., VO_2max_ and maximal aerobic velocity) and PT performance both in PPE and SG. In addition, the multiple regression analysis revealed that most of the variation in PT performance might be explained by the maximal aerobic velocity (84.5%) in PPE and the VO_2max_ (75.9%) in SG. These results seem to indicate that neuromuscular and anaerobic characteristics might have greater importance in PT performance when the WFF wore PPE [37].

All WFF completed the PT below the cut-score of 45 min (Table 2). Originally, this time was established based on the linear regression between PT total time and VO_2max_, associated with a minimum requirement for WFF of 45 mL·kg^−1^·min^−1^ [7]. Taking our data into account, a PT time of 45 min equated to a VO_2max_ of ~27 and ~34 mL·kg^−1^·min^−1^ for SG and PPE, respectively. This discrepancy was likely due to the fact that the subjects of this study walked at a self-selected velocity faster than the pace required to complete the test in 45 min. Indeed, the PT estimated VO_2_ (~39 and ~44 mL·kg^−1^·min^−1^ in SG and PPE, respectively) was substantially higher than that previously reported (22.2 mL·kg^−1^·min^−1^) [21]. The mean exercise intensity analysed in PPE and SG was higher (Table 2) than those observed during real wildland fire suppressions in professional WFF [9,38]. This was mainly because of the different character of the effort performed during the PT and wildfire firefighting (continuous vs. intermittent). However, when WFF performed the PT wearing PPE, the time spent above the RCT (~13 and ~4 min in PPE and SG, respectively) was similar to that observed in WFF during their deployments (~10 min) [9,38]. These results provide additional evidence on the role of PPE and self-selected pace in PT performance and how this fact might be of paramount relevance when assessing physiological readiness for work. A work readiness assessment should help evaluate WFF’s capacity to tolerate the highest work intensities and heat strain associated with wearing PPE [8,29]. It has been suggested that 15% of new WFF recruits who meet the PT pass scores would have difficulty performing their tasks while wearing PPE [12].

The present study presents some potential limitations. First, VO_2_ values were estimated. This might restrict the study validity since different factors such as circadian rhythms and thermal stress might have influenced the results [39]. Nevertheless, environmental conditions during the testing sessions and the fact that all tests were performed at the same time of day might have controlled the influence of these factors. Furthermore, it has been widely established that there is a strong relationship between HR and VO_2_ within subjects [22,40]. Second, the core temperature was not measured. Its measurement might have provided information about the subjects’ thermal strain and heat storage contribution to the physiological and perceptive response. In this regard, the HR reflected the combined load that the exercising muscles and thermoregulatory demands posed on the cardiovascular system. Therefore, the magnitude of the changes observed in this study between configurations may provide insight into the repercussions of wearing the PPE. Finally, the study did not consider female WFF, which may affect the obtained results. In this regard, additional research effort is required to determine if the findings reported in this study are applicable to females.

## 5. Conclusions

Findings from this study show that wearing complete personal protective equipment increases the professional WFF’s physiological strain, which translates into a significant reduction in PT performance. Our results highlight the requirement for the inclusion of personal protective equipment when assessing physiological readiness for work. In addition, it is advisable to perform the test by walking at the fastest possible self-pace. All this might contribute to achieving a high exercise demand, similar to those performed by the WFF in the hardest periods of work during wildfire suppression. Future studies should delve into the suitability of establishing a cut-off point of 45 min in this test.

## Figures and Tables

**Table 1:** Anthropometric characteristics and physiological values measured during the graded exercise test.

Variables	Mean ± SD
Age (years)	31.1 ± 3.2
Body mass (kg)	82.3 ± 12.2
Height (cm)	177.3 ± 7.2
Body surface area (m^2^)	2.0 ± 0.2
VO_2max_ (mL·kg^−1^·min^−1^)	55.8 ± 3.6
Maximal HR (beats·min^−1^)	185 ± 7
Maximal velocity (km·h^−1^)	16.9 ± 2.6
VO_2_ RCT (mL·kg^−1^·min^−1^)	45.4 ± 5.9
% VO_2max_ RCT	80.8 ± 4.3
HR RCT (beats·min^−1^)	172 ± 9
Velocity RCT (km·h^−1^)	14.3 ± 1.8
VO_2_ VT (mL·kg^−1^ min^−1^)	36.6 ± 5.8
% VO_2max_ VT	64.5 ± 4.9
HR VT (beats·min^−1^)	140 ± 7
Velocity VT (km·h^−1^)	10.3 ± 1.1

VO_2max_, maximal oxygen uptake; VT, ventilatory threshold; RCT, respiratory compensation threshold; %VO_2max_, percentage of VO_2max_ at which VT and RCT occur.

**Table 2:** Mean performance, physiological and perceptual responses (mean ± SD) during the Pack Test performed wearing short sports gear (SG) and personal protective equipment (PPE).

	SG	PPE	Cohen’s *d* (rating)
Pack Test completion time (min)	33.3 ± 3.9	37.2 ± 4.4 *	0.94 (large)
Heart rate (beats·min^−1^)	157 ± 9	169 ± 8 *	1.46 (large)
Percentage of maximal HR (%)	84.4 ± 4.4	89.7 ± 5.1 *	1.06 (large)
Estimated oxygen uptake (L·min^−1^)	3.2 ± 0.4	3.6 ± 0.5 *	1.06 (large)
Estimated oxygen uptake (mL·kg_total mass_^−1^·min^−1^)	33.1 ± 5.6	35.8 ± 4.9 *	0.51 (moderate)
Percentage of VO_2max_ (%)	61.0 ± 4.4	66.6 ± 4.2 *	1.34 (large)
Rating of perceived exertion	6.8 ± 0.6	7.4 ± 1.1	0.68 (moderate)
Walking cadence (steps·min^−1^)	155 ± 17	155 ± 17	0.00 (trivial)

* Significant difference (*p* < 0.05).

## Data Availability

The data presented in this study are available on request from the corresponding author. The data are not publicly available due to privacy restrictions.
